# Influence of heterospecifics on mesocarnivore behaviour at shared scavenging opportunities in the Canadian Rocky Mountains

**DOI:** 10.1038/s41598-023-34911-4

**Published:** 2023-07-07

**Authors:** Elicia Bell, Jason T. Fisher, Chris Darimont, Henry Hart, Christopher Bone

**Affiliations:** 1 Department of Geography, University of Victoria, PO Box 1700 STN CSC, Victoria, BC V8W 2Y2 Canada; 2 Department of Environmental Studies, University of Victoria, PO Box 1700 STN CSC, Victoria, BC V8W 2Y2 Canada

**Keywords:** Ecology, Behavioural ecology, Ecological modelling, Ecology, Behavioural ecology

## Abstract

In seasonal environments, the ability of mustelid species to acquire carrion—a dietary resource heavily depended upon—is driven by a collection local habitat characteristics and competition dynamics. In resource-scarce winter, sympatric mesocarnivores must balance energetic rewards of carrion with avoiding antagonistic interactions with conspecifics. We examined scavenging interactions among three mustelid species in the northern Canadian Rocky Mountains. Camera traps (n = 59) were baited with carrion during winter between 2006 to 2008. Spatial and temporal dimensions of scavenger behaviour (i.e., carcass use) were evaluated using a multi-model approach, which enabled us to recognize potentially adaptive behavioural mechanisms for mitigating competition at carcass sites. Best performing models indicated that carrion site use is governed by a combination of competition threats and environmental factors. A decrease in scavenging with increasing snow depth was observed across all species. Mustelids adopted a host of adaptive behavioural strategies to access shared scavenging opportunities. We found evidence that wolverine (*Gulo gulo*) and American marten (*Martes americana*) segregate in space but temporally tracked one another. Short-tailed weasel (*Mustela erminea*) scavenging decreased with greater site use by marten. Carcass availability across a spatially complex environment, as well as spatial–temporal avoidance strategies, can facilitate carrion resource partitioning.

## Introduction

Scavenging dynamics, shaped by the interplay between predation, competition and the external environment, represent an intricate lattice of energy acquisitions that perpetuate the food-web^1–4^. Carrion (i.e. remains of dead animals) is a unique form of detritus that exists as patches of concentrated energetic and nutrient value^1,5^. Occurrences of large ungulate carrion in forest ecosystems, for example, represent spatially distinctive “pulsed resources” that directly evoke behavioural responses by facultative scavengers and, in doing so, can alter the behaviour of species with which they interact^6^. In boreal forest communities, carrion represents a vital dietary component for many facultative scavengers, particularly in winter when energetic resources are scarce^7,8^.

Among vertebrate scavengers, mammalian mesocarnivores (small to mid-sized carnivorous species) are carrion consumers with unique capacity for influencing food-web dynamics^9^. Most mesocarnivores are opportunistic facultative scavengers. These taxa are frequently the dominant primary consumers of available carrion^10^, accounting for a high proportion (upwards of 88%) of carrion consumption^11^. In doing so, mesocarnivores expand the extent of energy and nutrient flows in food webs^10^.

Mesocarnivore scavenging patterns and their associative effects on food-web energy flows, are contingent on the ability of these species to utilize carrion resources. Regional availability and proportional dietary contribution of carrion to mesocarnivores is context-specific and often not well understood. Previous estimates suggest that carrion may represent > 30% of mesocarnivore diets^4^, with some larger mesocarnivores relying heavily on these resources^12^. Yet the ability of these species to locate and consume scavenging resources is governed by several factors related to competition and habitat structure^4,13,14^ Localized dynamic site attributes in relation to the physical landscape (e.g., landcover, topography), weather conditions (e.g. temperature, snow cover) and ecological community composition are important in this regard^2,3,14,15^. Landcover in particular (vegetation type, dominant forest canopy, hydric conditions) can influence habitat selection based on species-specific traits and is an important determinant of site use for these species in mountain landscapes^18,19,59,62^. The important role of habitat structure in partitioning unique assemblages of scavengers at carcass sites has been identified in numerous past studies (e.g. Refs.^2,8^). For example, decomposition occurs at different rates dependent on habitat structure and carcass exposure^37,38^, wherein rapid carcass decomposition associated with higher temperatures in open areas omit odors more readily, and olfactory cues appear to be the primary signal for carrion detection^22^. Carcasses can therefore be located and removed by scavengers more quickly contingent on their particular placement on the landscape^8^. However, for mesocarnivores, open areas may intensify scavenging competition, increase visual exposure to predators and reduce opportunities for escape among understory or arboreal features. Landcover can thereby change both the probability carrion will be discovered and the efficiency by which it can be consumed.

Weather conditions in seasonal environments can further alter scavenging dynamics by modifying carcass sites. During the cold season, past studies have observed a tendency for rate and frequency of scavenging by mesocarnivores to increase as ambient temperatures decline and have further recognized snow depth as a contributing factor to scavenger behavioural patterns^2^. Snow cover transforms carcass sites in three-dimensional space. Such altered structural complexity of habitats can induce behavioural changes owing to differentiation in thermoregulatory^23^, and hunting^24,25^ efficiencies. Indeed, snow depth has been recognized to alter mustelid distributions^26^, habitat selection^27,28^, competition dynamics^29^, and foraging behaviour^25^, and may provide mobility advantages for species able to utilize subnivean space. Evaluating habitat selection and snow depth—collectively referred to hereafter as environmental factors—in conjunction with competition strengthens the ability to decipher whether resource acquisitions are controlled by responses to competitive risk aversion or relates to some other aspect of overall habitat quality^13^.

Competition among species for carrion can also pose risk, which can be managed via behavioral strategies. Carcass sites generate rich localized feeding opportunities^7^ and can exist as points of intensified intraguild aggressions in the form of predation^30^ and competition^31^. Optimal foraging theory^34^ predicts that mesocarnivores will employ a scavenging strategy wherein energetic gains offset the potential costs of feeding on carrion. Individuals can minimize energetic costs associated with competition at carcass sites by selecting for carcasses in spatial locations that involve less risk of a competitive encounter, optimizing temporal downtimes of competitor activity or by limiting the overall extent (i.e. rate) of their resource use^32,33^. New behavioural insight into how mesocarnivores respond to one another in the presence of carrion resources can yield a more detailed understanding of scavenging dynamics in complex carnivore communities.

Drawing on these concepts, we asked whether patterns of winter scavenging behaviour change in relationship to external environmental stimuli (i.e., landcover and snow depth) and interference competition in a protected temperate mountain forest of western Canada. We focussed on a sub-group of cold-adapted mesocarnivores from the family Mustelidae with variation in body size, ecological traits and dietary niche overlap: short-tailed weasel (*Mustela erminea*, hereafter weasel), American marten (*Martes americana*, hereafter marten) and wolverine (*Gulo gulo)*. We used remote camera trapping^35^ and application of an integrated spatial–temporal research framework that combines regression models and time-to-event analysis to evaluate external factors governing carrion acquisition and identify behavioural tactics for mitigating interference competition. We use dualistic spatial–temporal approaches, which are needed to reveal species-specific behavioural coexistence strategies at fine spatial extents^36–39^. This work is situated at fine spatial (point location) and temporal (hourly) scales to gain fine-scale insights into the mechanisms of mesocarnivore coexistence.

Based on ecological theory, empirical evidence and inferences about competitive hierarchies based on body size, we weighed evidence for the relative impacts of competition versus surrounding environmental factors on scavenging behaviour to generate the following hypotheses. Firstly, we hypothesized that a combination of intraguild interactions and environmental site-level attributes would explain scavenger site use. We predicted that wolverine, the largest mustelid under investigation, will demonstrate competitive dominance over carrion sites, as evidenced by a lack of spatial or temporal response to co-occurring mesocarnivores. We predicted that smaller mesocarnivores, which are agile and capable of arboreal and subnivean mobility, would avoid wolverines in time, but not space. We also considered the effects of lynx (*Lynx canadensis*)—a larger mesocarnivore and potential predatory threat to all mustelids under investigation—to encompass a wider scope of mesocarnivore community dynamics. We hypothesized that wolverine will track Canadian lynx and hence co-occur in time and space, owing to periodic energetic rewards via carrion provisions as has been observed in Northern Europe^40^. By contrast, we expected lynx, proficient hunters of small to mid-sized mammals, to invoke fine-scale temporal avoidance responses in smaller mustelids, which may be able to avoid broader spatial exclusions via reactionary behavioural responses. Finally, we considered environmental factors, specifically landcover and snow conditions, as covariates to account for likely different habitat selection among species in a heterogenous landscape.

## Methods

### Study site

**Figure 1:**
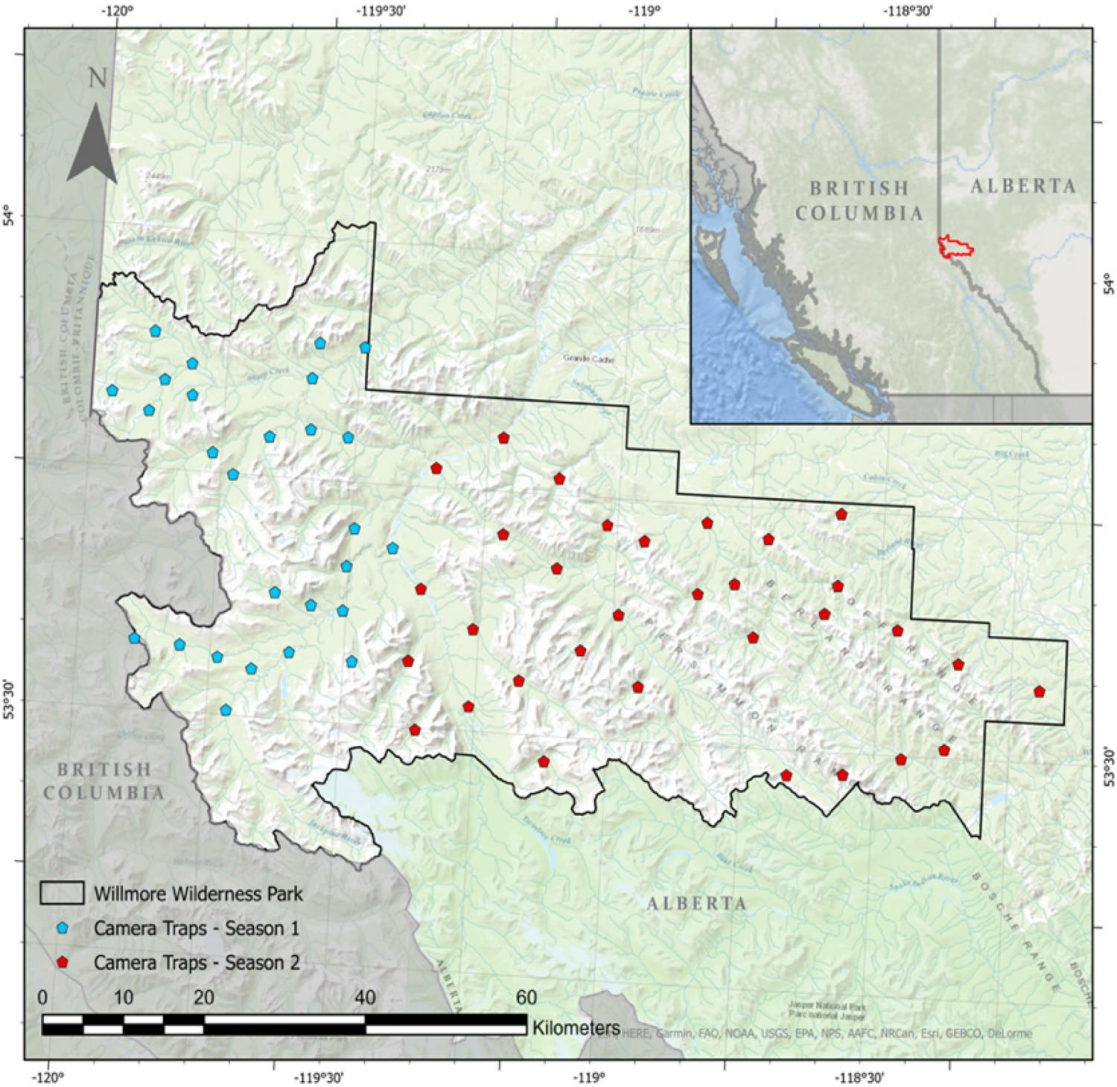
Willmore wilderness conservation area, Alberta, Canada. The WWP is bordered on its northern boundary by Kakwa Wildland Provincial Park and Protected Area in BC, and Kakwa Wildland Park in Alberta, and on the southern boundary by Jasper National Park and Rock Lake-Solomon Creek Wildland Park (Alberta). Camera trap data were collected over two consecutive winter seasons during 2006/2007 (period 1, n = 27, blue locations) and 2007/2008 (period 2, n = 32, red locations). Map created using ArcGIS Pro 2.8 (Data sources: Esri, HERE, Garmin, FAO, NOAA, USGS, EPA, NPS, AAFC, NRCan, GEBCO, DeLorme).

The Rocky Mountains span much of the length of the western Nearctic, and in Canada, runs the eastern edge of the Western Cordillera. Within this sampling frame, we selected our study area as one of the landscapes most highly protected from human development. The Willmore Wilderness Park (WWP) is a conservation area spanning approximately 4600 km^2^ in the Rocky Mountain region of Alberta, roughly 300 km northwest of the city of Edmonton on the Alberta-British Columbia border (Fig. 1). The WWP is bordered on its northern boundary by Kakwa Wildland Provincial Park and Protected Area in BC, and Kakwa Wildland Park in Alberta, and on the southern boundary by Jasper National Park and Rock Lake-Solomon Creek Wildland Park (Alberta). The region is heterogeneous with respect landcover and elevation, which ranges from 1200 to 2400 m above sea-level.

At higher elevations, the vegetation in the WWP consists of alpine meadows transitioning to subalpine fir (*Abies lasiocarpa*) at approximately 2000 m^41^. Sub-alpine conifer forests dominate the WWP landscape, consisting predominantly of Engelmann spruce (*Picea engelmannii*), white spruce (*Picea glauca*), lodgepole pine (*Pinus contorta*) and balsam fir (*Abies balsamea*), with stands of black spruce (*Picea mariana*) at lower elevations and river valleys^42,43^. The area experiences seasonal wildfires, resulting in burn sites that create open regenerating forest patches—mainly in the southwest and northwest areas of the park. Anthropogenic disturbance inside the WWP is restricted to recreational activities, equestrian activities, hiking, snowshoeing, backcountry camping, hunting, trapping and fishing^41,44^.

The WWP supports a diverse community of carnivores^43^. Large carnivores include grizzly bear (*Ursus arctos horribilis*), black bear (*Ursus americanus*), grey wolf (*Canis lupus*) and cougar (*Puma concolor*). The mesocarnivore community is comprised of coyote (*Canis latrans*), red fox (*Vulpes vulpes*), fisher (*Pekania pennanti*), lynx, wolverine, American marten and short-tailed weasel^16^. Wolverines represent the only focal species in this study currently of conservation concern in Canada. The species is described as “may be at risk” by the province, though detailed status is data deficient^45^, and is listed as Special Concern nationally^46^. The latest IUCN assessment for wolverines, taken in 2015, categorizes the species as ‘least concern’ but points to decreasing population trends globally^47^.

### Data

**Figure 2:**
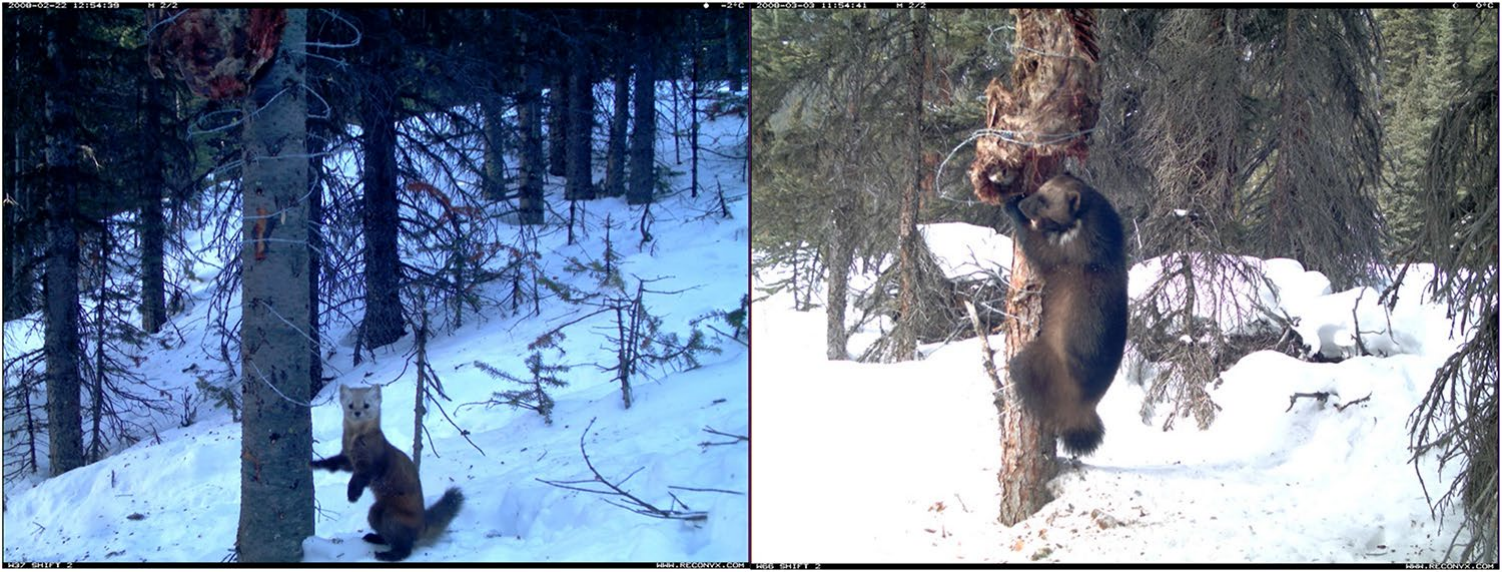
Camera trap images of marten (left, 22-02-2008) and wolverine (right, 03-03-2008) in the Willmore Wilderness Park, AB. Beaver carcass serving as a carrion scavenging opportunity is visible. Barbed wire at baited trap sites extracted DNA samples for wolverine as part of a study independent of this one.

This study utilizes camera trap data collected from a systematic sampling array (n = 66) deployed in the WWP repurposed from initial studies focused on wolverine spatial ecology^16,17,48^ and since used for several studies of mesocarnivore behaviour^49–51^. Reconyx infrared camera models PM30 and PM85 (Reconyx, Holman, WI, USA) were placed opposite a tree baited with a whole beaver carcass (Fig. 2), replenished monthly. Beaver offers a fat-rich reward in a nutrient-poor system. In addition, ca. 30 mL commercial scent lure (O’Gorman Long Distance Call, Broadus, MT, USA) was deployed at each carcass to enhance its detection by scavengers. Sites were deployed in a systematic sampling design approximately 5.73 km apart, according to a rectangular grid designed to capture heterogeneity experienced by mustelids in the Willmore. Cameras were placed *ca.* 1.5 m up the pole and set to high sensitivity, with no delay between triggers. Camera data were collected between late-December to early-March over two consecutive winters in 2006/2007 (period 1, n = 30) and 2007/2008 (period 2, n = 36; see details in Refs.^16,48^. Fifty-nine sites of the initial array were surveyed, while 7 were removed owing to mechanical failures. The study period covered a period during which all camera traps were consistently operational, totalling 70 days respectively for winter season 1 (2006-12-28 to 2007-03-08) and season 2 (2007-12-29 to 2008-03-08). The two seasons were combined to a single period by Julian date in order to evaluate the behavioural ecology of mesocarnivores during winter over the entire wilderness area, as in those companion studies.

### Analysis

#### ZINB regression models: estimating resource partitioning

Multiple lines of evidence were used to probe different key aspects of fundamental research objectives. To examine the influence of the surrounding environment and intraguild interactions on the relative intensity of site use by mustelid species, we used zero-inflated negative binomial (ZINB) regression model^52^. ZINB models involve a two-tiered process that estimates the degree of influence of predictor variables on both species presence and the rate at which camera sites are used and assume zero-inflation in the data is in part owing missed detections when a species was present^53^. The response metric is the photographic capture rate (CR) for focal species at each trap location over consecutive 5-day sampling intervals spanning the duration of the study period (n = 826 sampling occasions). Weasels were detected in the second winter season only, therefore CR data represent the 2007/2008 winter (n = 448 sampling occasions) for this species alone. Based on our hypotheses we included lynx as a competition variable in ZINB models in addition to sympatric mustelids.

To ensure data represented independent site visitation events of unmarked species, we removed successive captures of the same species occurring within 10 min, similar to procedures followed in other camera trap data analyses (for example ^54^). Repeat captures of the same species exceeding 10 min were considered indicative of either prolonged site usage by the same individual or the appearance of a different individual. When multiple individuals were captured during a single capture event, each individual was counted as an independent data point. We reviewed overall CR of scavengers at baited traps and chose 5-day sampling occasion lengths; these are expected provide enough time for focal species time to investigate their home range for scavenging opportunities and to react to heterospecifics in their territory.

Zeros in camera trap survey count data are common, arising anytime a focal species is not detected over a defined sampling occasion (Supplementary Fig. S1). Attractants typically serve to increase carnivore detections at camera sites, thereby reducing the severity of zero-inflation the data^55^. We assessed CR data for zero-inflation and overdispersion to select the most suitable modelling design for analyzing these data—i.e. a ZINB, a zero inflated Poisson model (ZIP), or a general linear model for Poisson or negative binomial distributions (GLM-P, GLM-NB)—and strengthen ecological inferences^56^. Mustelid CR datasets were tested for overdispersion using the Pearson dispersion statistic^57^. Inspection of zero-inflation of count data was performed by the Vuong test commonly used to look for zero-inflation^58^ (but see Ref.^59^).

ZINB is comprised of two distributions: (1) a binomial logistic regression that accounts for the excess zeros, and (2) a Poisson or negative binomial count process that examines CR. We considered five explanatory variables for both ZINB processes: three sympatric scavenger competition variables (accounting for each respective competitor) and two environmental factors—snow depth and landcover. Competition metrics are defined as competitor presence/absences for the binomial process and competitor CR per sampling occasion for the count process. We observed only low correlation among competitor covariates (Supplementary Table S1). Weak correlation (all Tau statistics < 0.15) existed in independent capture data for Wolverine–Marten, Marten–Weasel and Marten–Lynx. All variables were thus retained on the basis of their known biological significance. Odds ratios (i.e. exponentiated regression coefficients) offer a measure of the strength of association between the predictor and outcome.

To examine the role of vegetative cover on frequency of site use, landcover classifications were derived from the Alberta Satellite Land Cover (ASLC) raster produced by the Government of Alberta^42^ and classified post hoc as either open (e.g. herbaceous areas, wetlands, grassy meadows) or closed (e.g. conifer or mixedwood forest) canopy at the site level (Supplementary Table S2).

Owing to the observed influence of snow depth on mustelid behavioural ecology and distributions we further examined daily snow depth as a predictor of localized scavenging behaviours. As snow depth is dynamic with respect to time, this factor further allowed us to account for seasonal differences between the two study seasons. Snow cover is persistent across much of the WWP during the winter months with variability in depth correlating with elevation, terrain ruggedness and seasonal climate variability. Daily snow depth analysis measures were performed by Brown and Brasnett^60^ for the same study period, using a combination of in-situ observations (i.e., snow measuring rod sensors) and a snow accumulation and melt model.

To assess the relative strength of environmental factors against the influence of competitive interactions among facultative scavengers, we evaluated a set of three candidate models that included environmental factors exclusively, intraguild interactions exclusively, and a combined model that incorporated both sets of parameters. Akaike’s Information Criterion (AICc) scores—wherein we considered models with ΔAICc < 2 as having different explanatory power—and normalized AICc weights were used to rank candidate models (sensu ^61,62^), and best performing models were identified based on the lowest AICc score.

### Temporal spacing analysis

A temporal spacing analysis was employed to understand whether spatially co-occurring mustelid species react to one another through immediate, reactionary temporal avoidance. Mustelids are equipped with sensory capacities for detecting the recent presence of species that represent a predatory or competitive threat^63,64^. An observed time-to-event (TTE_obs_) dataset was compiled to test whether fine-scale temporal patterns of mustelid encounters at camera sites differed from what would be expected under random conditions. An ‘event’ is characterized as the photographic capture of a focal species, hereafter referred to as species B (SpB) following the capture of a heterospecific, designated species A (SpA), at a specified spatial location. It follows then that the capture time of the SpA serves as a reference point with respect to time (t = 0). In instances where multiple successive captures of the same species occurred, time-to-event (hours) was calculated by observing the time difference between the last capture of the first species and the first capture of second species. We assumed that direct interactive processes were likely to occur inside of this timeframe, as avoidance or attraction occurring over broader (e.g. monthly) temporal scales is more likely to be related to niche partitioning processes^43^ than immediate perceived threat. Some sympatric carnivores have been shown to coexist by practicing fine-scale spatiotemporal avoidance, thereby avoiding suppression at the habitat or landscape scale^65^. The study interval chosen is designed to align with the duration of sampling occasion used in the ZINB analysis so that we might draw comparisons on the manner in which the mechanisms of coexistence operate.

We generated randomized capture data for each focal species for 1000 iterations to compare observed TTE_obs_ data to indiscriminate encounter data. To accomplish this, we randomly selected a spatial location by choosing a unique camera trap location where the species of interest had been observed at least once over the study period. Next, we selected a new capture date at random from the survey period, where if the camera ID number identified in step 1 fell between 1 and 30, we selected dates from winter season 1 (2016/2017). Alternatively, if the camera ID was between 31 and 66, we selected from the date ranges for winter season 2 (2017/2018). We then select a new time by sampling the diel activity pattern probability density function of the corresponding focal species. The new random capture data were integrated with the observed data for the reference point, and we then calculated the TTE between species pairs and removed any events that exceeded 120 h (5-days). In the observed TTE (TTE_obs_) distributions, event data points were removed if a third-party animal with potential to influence the behaviour of focal species arrived between the reference point and the event thereby possibly altering the timing of the event. Under this scenario, as time increased so too did the possibility of an intermittent site visit by another animal.

By examining the TTE_obs_ dataset, we noted that between 68 and 91% of events recorded for species pairs were represented by “true events” (i.e. no intermittent species occurrence), with an average of 78%. To emulate this aspect of TTE data, we applied a probability density function with exponential decay to the new TTE_r_, such that the probability of selecting an event decreased with increased time-since measure, then randomly chose 80% of those data without replacement to generate the final TTE_r_. Finally, we compared mean values of the TTE_r_ and TTE_obs_ datasets using a two-sample Mann–Whitney U test^66^, a standard test for examining differences between two groups with skewed distributions. Where the p-value of the test is less than the significance level alpha = 0.05, we can conclude that the average TTE_obs_ significantly differed from the TTE_r_.

### Animal ethics statement

All experimental protocols were approved by the Alberta Research Council’s Animal Care Committee. All methods were carried out in accordance with the regulations outlined under Alberta’s Wildlife Act, licensed by the Province of Alberta under their Research and Collections Permit. The study was carried out in compliance with the ARRIVE guidelines, and the guidelines required by the Canadian Council on Animal Care (CCAC).

## Results

Wolverines were detected across 51 camera trap sites (n = 1080). Marten occurred at 53 camera-trap sites, the most captured mesocarnivore at carcass sites by an appreciable margin (n = 12,599). Weasel occurred at 15 trap locations (n = 342), exclusively during the winter 2007/2008 season. Lynx were observed at 11 unique camera locations (n = 305). Other competitors, cougar (n = 67), wolves (n = 31), red fox (n = 194) and fishers (n = 147), occurred at low frequancies.

### Spatial interactions and habitat use

The presence of competitors—together with landcover heterogeneity and snow depth—best explained mountain mustelid spatial occurrence. ZINB were found to be the best suited models for marten and wolverine datasets, whereas a NB-GLM was more suitable for the weasel distribution. In the case of weasel count data, the negative binomial general linear model resulted in a comparable dispersion statistic to that of the ZINB model. The Vuong statistic revealed that the NB-GLM performed better for weasel count data and thus represented a more suitable model for these data (Supplementary Table S3).

Results from the ZINB and NB-GLM model selection revealed that spatial patterns were best supported by a combined model that included both habitat and competition predictor variables for wolverines (AICw = 0.997), marten (AICw = 0.98), and short-tailed weasels (AICw = 0.86) (Table 1). For wolverines, the environment-only and competitor-only models possessed similar explanatory power (ΔAICc < 2), with the combined model outperforming both (Table 1a). For marten and weasels the environment-only models performed better than the competitor-only models but were both eclipsed by the combined model (Table 1b,c).

**Table 1:** ZINB/NB-GLM model selection for (a) wolverine, (b) American marten, and (c) short-tailed weasel spatial distributions during winter in the Willmore Wilderness Park, Alberta. ZINB/NB-GLM models are ranked according to model weight associated with AICc values. Parameters are designed to create representational models for: competition related factors (M1), habitat character factors (M2) and a full model consisting of combined habitat-competition factors (M3).

Model number	Predictor variables		
M1 (k = 3, df = 7)	Mustelid no.1 + mustelid no.2 + Lynx		
M2 (k = 2, df = 9)	Landcover + Snow depth		
M3 (k = 5, df = 13)	Mustelid no.1 + mustelid no.2 + Lynx + Landcover + Snow depth		

a. Wolverine model rank	$\Delta$ AICc	Weight	Log-likelihood
M3	0.00	0.997	− 1028
M1	12.47	0.002	− 1041
M2	13.39	0.001	− 1039

b. Marten model rank	$\Delta$ AICc	Weight	Log-likelihood
M3	0.00	0.981	− 2638
M2	7.93	0.019	− 2648
M1	158.83	0.000	− 2721

c. Weasel model rank	$\Delta$ AICc	Weight	Log-likelihood
M3	0.00	0.856	− 367
M2	3.56	0.144	− 368
M1	26.40	0.000	− 382

Presence of wolverine was significantly impacted only by that of marten (p = 0.015, $\beta$ = − 0.582), where the likelihood of wolverine presence over 5-day periods was 56% more likely at sites that lacked martens (Table 2a). By contrast, the CR of wolverine increased modestly with marten CR (p = 0.028, $\beta$ = 0.01), whereas the rate of lynx site use showed a negative association with wolverine CR (p = 0.03, $\beta$ = − 0.119). Wolverine presence was highest at sites with low relative snow depth (p < 0.001, $\beta$= − 0.011). Similarly, CR of wolverine at scavenging opportunities decreased with increased snow depth across the ~ 20 cm to 200 cm range. Landcover type did not impact wolverine spatiotemporal distributions or CR (Table 2a).

**Table 2:** Zero inflated negative binomial model outputs for capture rates of (a) wolverine, (b) American marten and (c) short-tailed weasel. Table values include regression coefficients estimate ($\beta$), exponentiated regression coefficients (exp($\beta$)) that represent the odds ratio, standard deviation (SD) and p-value (significance level = 0.05, indicated with ‘*’).

(a) ZINB Model M3: Wolverine Zero-inflation model coefficients (binomial with logit link)	Estimate	Exp ($\beta$)	SE	p
(Intercept)	1.521	4.577	0.341	< 0.001*
Marten	− 0.582	0.559	0.265	0.015*
Weasel	− 0.218	0.804	0.365	0.115
Lynx	− 9.439	7.96 e−05	82.918	0.909
Landcover (open)	0.070	1.072	0.309	0.765
Snow depth	− 0.011	0.989	0.004	< 0.001*
Count model coefficients (negative binomial with log link)				
(Intercept)	1.554	4.730	0.273	< 0.001*
Marten (CR)	0.010	1.010	0.004	0.028*
Weasel (CR)	0.190	1.210	0.120	0.550
Lynx (CR)	− 0.119	0.888	0.055	0.030*
Landcover (open)	0.072	1.075	0.243	0.822
Snow depth	− 0.009	0.991	0.002	0.001*

(b) ZINB Model M3: Marten Zero-inflation model coefficients (binomial with logit link)	Estimate	Exp ($\beta$)	SE	p
(Intercept)	1.898	6.673	0.341	< 0.001*
Wolverine	− 0.504	0.604	0.253	0.047*
Weasel	0.275	1.317	0.324	0.396
Lynx	0.964	2.622	0.502	0.055
Landcover (open)	− 1.526	0.217	0.406	< 0.001*
Snow depth	− 0.032	0.968	0.004	< 0.001*
Count model coefficients (negative binomial with log link)				
(Intercept)	2.183	8.873	0.154	< 0.001*
Wolverine (CR)	0.020	1.020	0.014	0.148
Weasel (CR)	− 0.037	0.964	0.049	0.449
Lynx (CR)	− 0.137	0.872	0.056	0.013*
Landcover (open)	− 0.163	0.850	0.126	0.195
Snow depth	0.008	1.008	0.001	< 0.001*

(c) GLM-NB M3: Weasel	Estimate	Exp ($\beta$)	SE	p
(Intercept)	3.295	27.0	0.622	1.18 e−07*
Wolverine	0.031	1.03	0.042	0.463
Marten	− 0.059	1.06	0.018	9.59 e−4*
Lynx	0.022	1.02	0.033	0.510
Landcover (open)	0.165	1.18	0.390	0.673
Snow depth	− 0.061	0.94	0.010	2.92 e−09*

Marten presence was highest in closed-forest habitats (p < 0.001, $\beta$ = − 1.526) with low relative snow depth (p < 0.001, $\beta$= − 0.032). However, at locations where marten occurred, CR increased by nearly 1% for every 1-cm rise in snow depth (p < 0.001, $\beta$ = 0.008). Habitat type in particular was highly influential in determining marten presence, where it decreased by 78% in open sites relative to closed forest canopy (Table 2b). Landcover type did not alter marten CR.

Competitive interactions appeared to negatively influence both marten presence and rate of site use. Marten presence was approximately 40% lower at sites that had been occupied by wolverines inside the same 5-day period. The presence of lynx showed no association with marten presence, however, increasing intensity of lynx site use reduced marten CR by a factor of 0.87 or about 13% (Table 2b).

The relative frequency of weasel site visits decreased with increased snow depth (p < 0.001, $\beta$ = − 0.061). Weasel CR was also sensitive to the frequency of site use by competitors, declining with increasing counts of marten (p < 0.001, $\beta$= − 0.059). Weasel CR was not associated with canopy closure (Table 2c).

### Temporal spacing analysis

**Figure 3:**
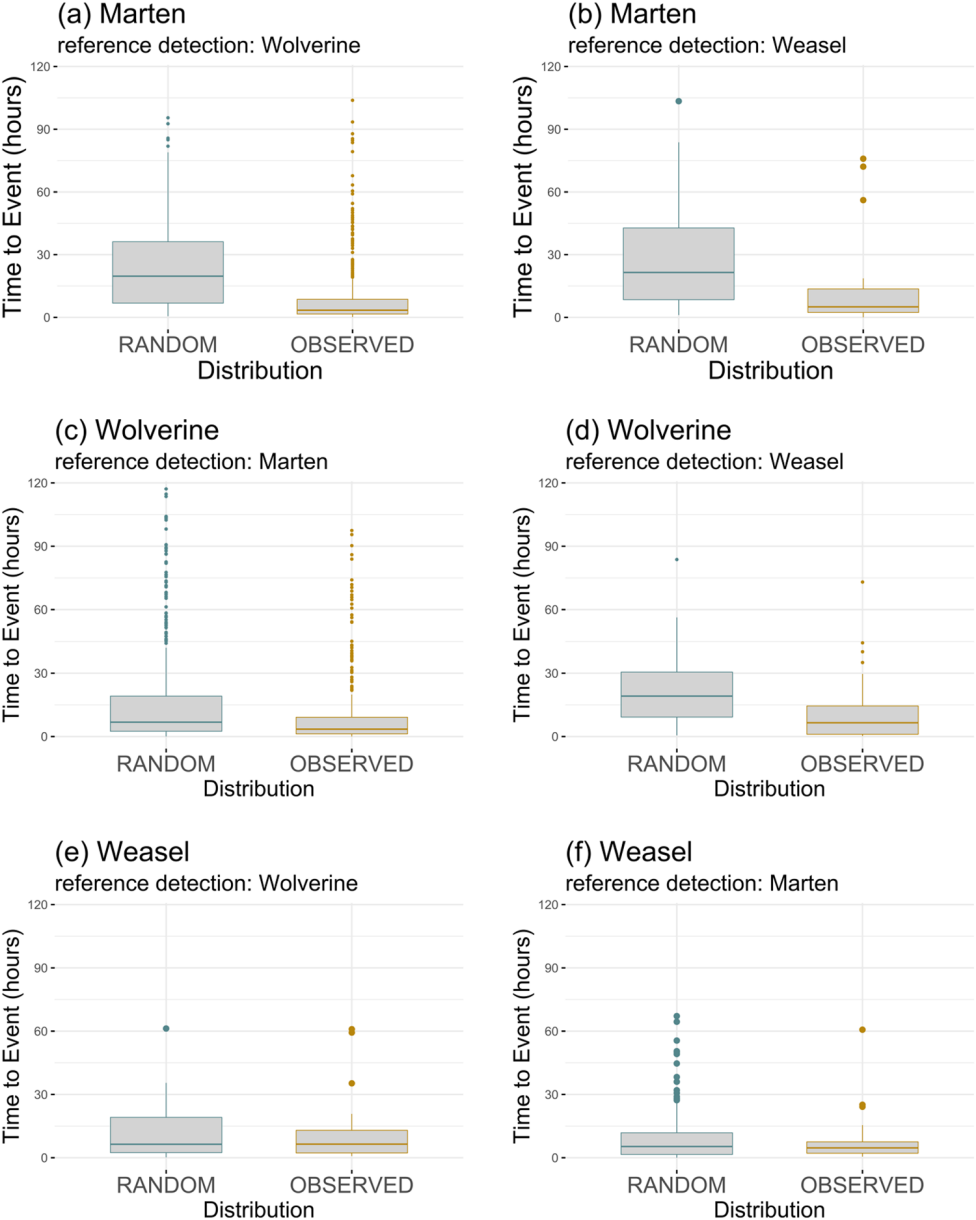
Temporal spacing box plots representing results of Wilcoxon rank sum test for 2-group comparison of average time to event (TTE) according to random (TTE_r_) and observed (TTE_obs_) time-since distributions, following a competitor detection, for all focal species pairs. Reference detections represent the competitor that visited the resource first. Median time-since measures for TTE_obs_ were significantly different from TTE_r_ (plots **a**–**d**) where p < 0.05 (Supplementary Table S4).

Wolverines temporally tracked both martens and short-tailed weasels. Wolverine temporal detections relative to martens and weasels differed from random (p < 0.001 and p = 0.002 respectively), appearing at carrion sites following smaller mustelid species in lesser time on average than random (Fig. 3). Observed time-to-event of wolverine following weasel (mean = 12.3 h, Supplementary Table S4) was nearly half expected mean time-to-event (mean = 22.2 h, Supplementary Table S4), after accounting for patterns in wolverine diel activity. We observed similar patterns of apparent temporal attraction by martens, which visited carrion sites subsequent to co-occurring mustelids: wolverine (p < 0.001) or weasel (p < 0.001) (Fig. 3). In both cases, marten appeared at a site within 12 h (on average) of a potential competitor, less than half the anticipated amount of separation time for wolverine (mean = 24.9 h, Supplementary Table S4) and weasels (mean = 29.4 h, Supplementary Table S4). Weasels neither avoided nor showed evidence of attraction towards wolverine (Fig. 3). Average time until weasel appearance did not differ significantly from expected when they followed larger mesocarnivores, wolverine (p = 0.96) or marten (p = 0.75).

## Discussion

In winter mountain landscapes, competing mesocarnivores used species-specific avoidance of potential competitors that included a mixture of spatial and temporal strategies. As predicted, these patterns also varied with landcover and snow depth conditions. However, in some cases where spatial segregation occurred, intensity of site use in areas of sympatry was either unimpacted, or increased, as in the case of wolverine relative to marten. Moreover, when wolverines and martens did co-occur in time and space, they appeared to track each other’s activity, occurring at a scavenging opportunity in half the time as expected following appearance of the competitor. We interpret this as capitalizing on the presence of a competitor to find foraging opportunities, and a potential strategy to mitigate resource loss to competitors.

### Facultative scavenger species interactions

After accounting for differences in environmental attributes, feeding behaviours of wolverines were markedly influenced by intraspecific interactions. Despite the supported prediction that wolverine would dominate carcasses and suppress smaller mesocarnivores^67^, wolverine presence was negatively associated with marten presence, and vice versa. This may be due to what Murrell and Law^68^ described as heteromyopia, in which interspecific competition plays out over shorter distances relative to intraspecific competition. Under this scenario, strong intraspecific competition within the more abundant species reduces local density, thereby creating spatial openings occupied by the lower-density competitor^69^. While martens are competitively inferior to wolverines with respect to weaponry and body size, they may exert strong competitive forces indirectly through exploitative competition—a distinct form of competition that occurs when one species depletes a common resource thereby limiting that available to their competitor. While we could not directly measure intraspecific competition and as Blanchet et al.^70^ noted, species co-occurrence is not a de facto measure of species interactions, heteromyopia offers a strong theoretical explanation for our observations and supports evidence of co-occurrence.

We suggest heteromyopia may help to reconcile findings that, while marten and wolverine segregated in space, the intensity of wolverine site visitation rate increased in relationship to martens’ site visits. Inherent in heteromyopia (and other theories of spatial competition) is that a higher-density but subordinate species (marten) can win in exploitation competition over lower-density but dominant species (wolverine), except within competition gaps^69^. In heterogenous environments, heteromyopia can thus manifest as segregation between sympatric species at a localized scale and higher average densities (aggregation) of the more common competitor relative to the rarer one^48,68,69^. Marten was by far the most frequently detected mustelid species in this survey, with independent captures surpassing wolverine by more than ten-fold. Moreover, Fisher et al.^48^ found evidence that marten tend to aggregate where they occur inside this mountain landscape. Thus, although more extensive multi-scaled analysis would be required to fully confront this hypothesis, patterns we observed suggest a heteromyopia process may be at play for these two species.

We interpreted the increased wolverine site use (i.e. prolonged feeding or frequent recurrences) at carrion sites that marten also used as potential evidence for kleptoparasitism. Wolverine often arrived at carcass sites very soon after marten, or even while a marten remained present (10% of all wolverine events occurred less than 30 min following a marten). Almost all mustelids use scent signaling to communicate and olfactory cues from prey are important determinants of foraging efforts^71^. Thus, wolverine may exploit martens by tracking their scent markings to locate carcass sites already discovered by the smaller mustelid, and to mitigate resource losses to this numerically superior subdominant competitor.

By comparison, the intensity of marten site use was unimpacted by wolverines. Moreover, marten frequently used sites the same day as wolverines. These patterns of behaviour suggest a net energetic gain for marten despite any immediate threat of a wolverine confrontation. Wolverine possess physiological adaptations for feeding on bone and frozen carcasses^72^. Carcass openness has been described as a highly important determinant of carcasses use time and accessibility to scavengers^20^. Selva et al.^20^ found that wolves played a vital role in altering bison carcasses to facilitate feeding accessibility by other carnivores through progressively opening previously inaccessible regions of the body cavity. Wolverine may offer a similar service to subdominant competitors, explaining martens’ temporal tracking of wolverines.

Furthermore, martens in the WWP maximized access to carcasses through adaptive competitor-specific behavioural tactics. Rather than avoiding locations used by lynx, marten reduced their rate of scavenging in lynx high-use areas. This competitive interaction contrasts that described in Spain where reintroduced Iberian lynx was generally associated with spatial displacement of sympatric mesocarnivores^73^. Lynx is an occasional predator of marten, and although predation is not thought to be substantial to impact marten populations^74^, our findings suggest that they may limit the frequency at which marten are able to utilize a shared resource.

In a broad spatial context, lynx site occupancy had no effect on wolverine spatiotemporal presence. This aligns with past research by Mattisson et al.^12,40^; although wolverine scavenge a high proportion of Eurasian lynx (*Lynx lynx*) reindeer kills (68%)—almost half of which remained occupied by a lynx on the arrival of wolverine—there was no evidence of spatial or temporal attraction or avoidance between these two species. Our findings therefore challenge the assumption that wolverine track and scavenge ungulate kills by Canadian lynx^47^, while also suggesting that wolverine do not actively avoid lynx at the landscape scale, at least in this Nearctic mountain system. These findings are further supported by Chow-Fraser et al.^49^, who found no evidence for facilitation by lynx in relation to wolverine. Canadian lynx is a specialized predator of snowshoe hare^75,76^, a species abundant in the study area and also preyed upon by wolverine^72^. It is possible that in the absence of sufficient potential for ungulate carrion availability, lynx in the northern Rocky Mountains exert little influence on wolverine with respect to food provisions. Where wolverine and lynx presence overlapped spatially (9 of 66 camera locations), wolverines did however alter their rate of scavenging in response to increasing site use by lynx, suggesting that wolverine primarily reduced their risk of exposure to lynx encounters via fine-scale avoidance tactics. Comparatively, Klauder et al.^67^ found that wolverine in Alaska expressed very low vigilance even under the continued presence of competitively superior wolves, on occasion yielding carcasses only under direct confrontation. Our findings similarly suggest that the only a more immediate threat of competition with lynx was enough to outweigh the effects of continued access to energetic gains. The fearless tendency of wolverines at scavenging sites appears to have resulted in a lack of segregation at broader spatial extents.

### Influence of habitat and snow depth on scavenging potential

Competition plays out in a spatially complex environment. We show that landcover and snow depth both affect mustelid occurrence at scavenging sites in addition to species co-occurrences. Notably, wolverine and weasel used open and closed forested sites indiscriminately, inconsistent with typical patterns of habitat selection for these species (e.g. ^77,78^). Evidence suggests that open habitats may foster competition among carnivores across an array of ecosystems possibly because prey carcasses are more readily discovered in those environments^21^. Scents emitted by carcasses may travel easily and more rapidly in open areas relative to closed habitats^79^. Mustelids are efficient at locating food remains^25^ and are likely to detect and locate these sites efficiently, conceivably increasing competitive interactions with one another. This is substantiated by findings in Germany that mesocarnivores appeared first at carrion sites nearby open meadows^8^ and in Poland where carcasses in forested relative to open areas were depleted over lengthier periods^20^. Differentiation in habitat use can serve to foster co-existence, however evidence found here suggests that, at carrion sites, mustelid interactions could intensify by breaking down habitat niche partitioning.

Snow depth can further influence movements and incur metabolic costs to mustelids, which can negate gains made from foraging at carcasses^80–82^. Martens are highly adapted to mobility over deep snow^83^ though under conditions of high snow accumulation, they also use subnivean cavities as rest sites to reduce heat loss^23^. Reduction in marten presence in response to deepening snow is likely to partially reflect increased periods beneath the snow owing to the thermal efficiencies of these areas under harsh winter conditions as well as the threat of being exposed to predation and competition^23^.

The role of snow in wolverine behavioural ecology and indeed, interspecific interactions, remains unclear. Wolverines are a cold-adapted species, possessing morphology that allows them to efficiently travel across snowy environments, yet there is a lack of consensus in existing literature surrounding wolverine snow-reliance (see review by Fisher et al.^27^). Snow cover persistence and depths are widely held to be highly important to wolverine spatial distributions, food caching and reproductive den site requirements^27,84–86^. By contrast, we found that increasing snow depths reduced wolverine presence at carcass sites and frequency of visits. However, our results indicate a weak association between snow depth and overall patterns of carrion site usage by wolverines (odds ratios for both CR and presence/absence ZINB model components < 0.001). Habitat characteristics such as landcover and food availability can outweigh the effects of snow on wolverine space use^18,87^. Similar to landcover, though our analysis clearly revealed that snow depth plays a role, heterospecifics make a significant contribution to scavenging behaviours with larger effect sizes.

### Limitations

Remaining unexplained variability in mustelid site usage may be due to the presence of other competitors—cougar, wolves, red fox, and fishers—we omitted, owing to a lack of data. This could potentially hinder our interpretations of mustelid scavenging strategies in relation to competition. For instance, fishers and competitively subordinate martens, co-occur and engage in intraguild interference competition across their range^29,88^ that can result in competitive exclusion^89^ and spatial segregation during winter^90^ as has been observed in the WWP^48^. In another example, wolverines have been known to track wolves due to opportunities for carrion provisions^67^. Thus, including fishers, wolves and other species may improve our understanding of fine-scale mustelid spatial–temporal distributions. However, the fact that some species (cougars and wolves in particular) occurred very infrequently support that the scavenging competition relationships we examined reflect the major competitors that species must contend with in these landscapes. Thus, although this research shows a partial view of scavenging dynamics, the results we obtained are novel and hold value in furthering understandings of competitive interactions between species that rely heavily on scavenging in harsh environmental conditions.

There might have also been limitations related to the state of the carcass (i.e. phase of consumption/decomposition). Carcass condition has previously been found alter carcass use by scavengers^2^ and is likely also an important determinant of immediate decisions to scavenge in relation to competition at this scale of investigation. Bait rewards at camera sites in the Willmore were replenished monthly, however, days-since measures of carcass placement alone is not a reliable estimate of carcass condition. Consumption and decomposition occur at markedly different rates contingent on site-specific environmental conditions (e.g. open versus closed habitat)^20^. Any bias may therefore have resulted from fluctuating degree of energetic payoff at carrion sites.

## Conclusions

Mustelids in this northern mountain system demonstrated fine-scale behavioural adaptations and reactionary temporal avoidance strategies that would facilitate sympatry and maximize energetic rewards. In multi-carnivore communities, interference competition—defined by direct acts of aggression that encompass harassment, non-consumptive or consumptive killing, kleptoparasitism and infanticide—is widespread, often manifesting in altered foraging behaviour and habitat use^39,65,91^. The extent of interference competition observed among carnivores is primarily determined by the extent of competition over resources^9^, and scavenging can represent a pathway of direct competitive encounters^92^. Thus, while carrion is unique in that it offers energetic gains without the expenditures required to capture and overpower prey, these resources can pose a heightened risk of mortality for mesocarnivores^4,30^. However, carrion provisions during winter environmental conditions can stabilize annual food uptake and is thus a vital dietary component for many facultative scavengers^93^. At the level of the individual, carcass feeding behaviour is based on outcomes of energetic cost–benefit analyses^67^, which are subject to influence by external environmental factors^2,15,94^. We uncovered the likely mechanisms by which facultative scavengers minimize costs associated with competition to share in benefitting from a valuable winter resource.

**Figure 4:**
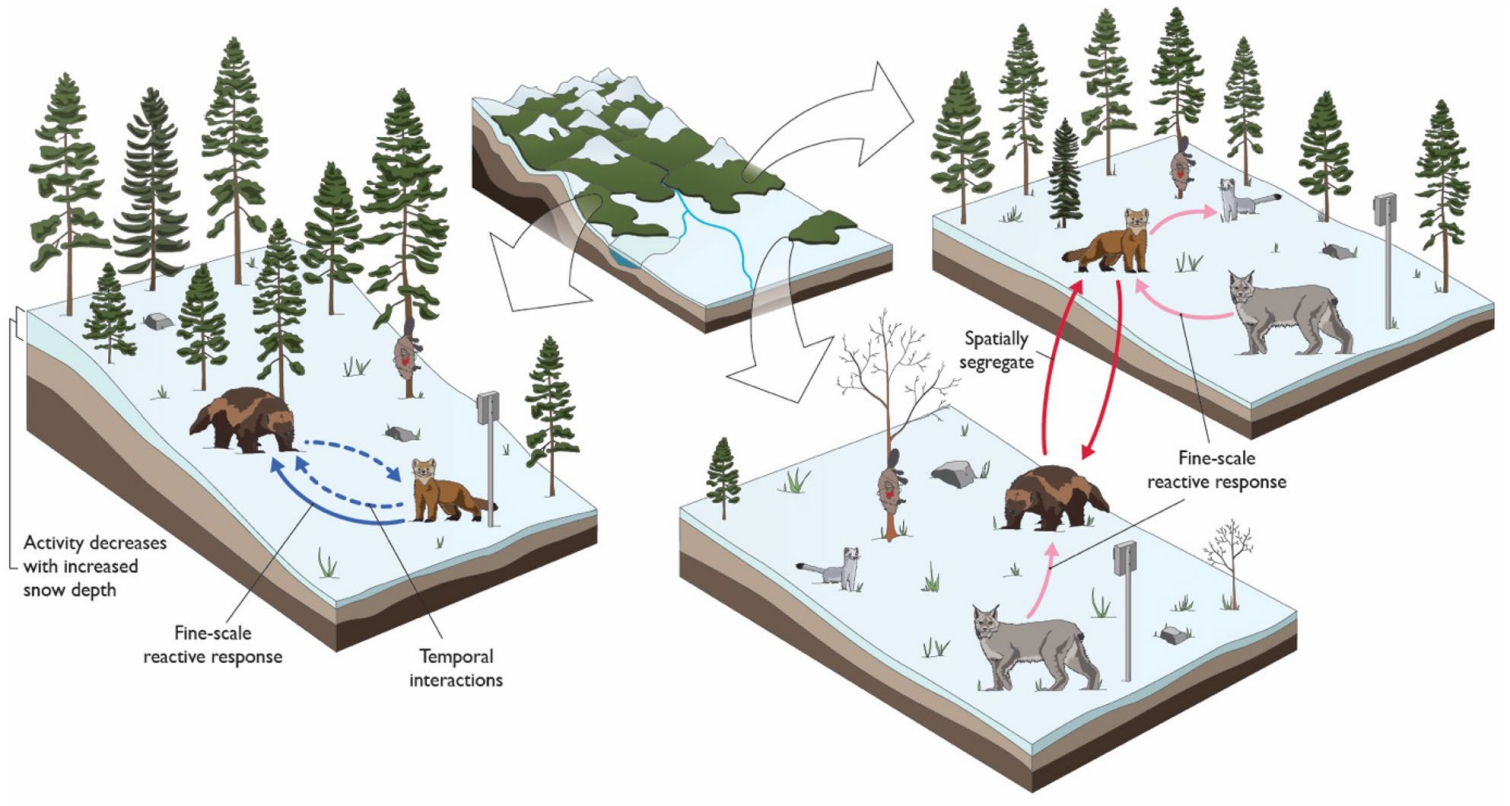
Mustelids are subject to influence by environmental features and competition at winter scavenging opportunities. Species interact with their surroundings at carrion sites using various adaptive mechanisms for optimizing foraging uptake, including (1) site-level spatial segregation (solid red/dark blue arrows), as well as (2) fine-scale spatial reactionary responses (solid pink/light blue arrows), and (3) fine-scale temporal reactionary responses (dotted pink/light blue arrows). Arrows indicate directionality and nature of the effects. Negative relationships are represented in red for site-level spatial segregation and pink for fine-scale spatial and temporal responses. Positive associations are represented by blue arrows.

Competing species partition both space and time to acquire limited resources such as winter scavenging opportunities (Fig. 4). Species that segregate in space, and over the time-scale of weeks, to avoid (or as an outcome of) competition nevertheless aggregate in hours and days, which we suggest is a behaviour adopted to mitigate the risk of resource loss given the intrusion of a competitor.

Improved insight into carrion use by facultative scavengers and the associative factors that shape carrion acquisition for those species will deepen our understanding of broader ecosystem processes. Competition over large mammal carcass sites in the boreal forests and mountainous landscapes in western Canada may be especially pronounced, especially in winter when resources become more limited. Key findings revealed that mustelids optimize a mixture of behavioural tactics to combat energy losses associated with the interplay of competition factors and environmental conditions. Reactionary spatial and temporal responses to intraguild competitors at carrion sites appeared dynamic and subject to localized site conditions. Microhabitat composition and differences in attraction/avoidance techniques of mountain mustelids appeared to alter site visitations and may govern accessibility to carrion in a multi-carnivore landscape.

As climate change threatens to modify existing structures of scavenging community composition and dynamics globally^5,95^, scavengers, which are limited by competition or depend heavily on carrion resources, may experience disproportionate impacts from such changes. Understanding the factors that drive access to these ephemeral resources can provide valuable information for anticipating impacts of facultative scavengers under changing climate conditions in the boreal forests of western Canada and elsewhere.

## Supplementary Information

Supplementary Information.

## Data Availability

The datasets generated during and/or analysed during the current study are available from the corresponding author on reasonable request.
