# Supplementary material for: Influence of heterospecifics on mesocarnivore behaviour at shared scavenging opportunities in the Canadian Rocky Mountains
Source: Sci Rep. 2023 Jul 7;13:11026. doi: 10.1038/s41598-023-34911-4 (PMC10329011; doi:10.1038/s41598-023-34911-4)
Supplement: Supplementary file 1 — Supplementary Information. [file 41598_2023_34911_MOESM1_ESM.docx]

**Supplementary Information: Influence of heterospecifics on mesocarnivore behaviour at shared scavenging opportunities in the Canadian Rocky Mountains**

Elicia Bell*, Jason T Fisher, Chris Darimont, Henry Hart & Christopher Bone


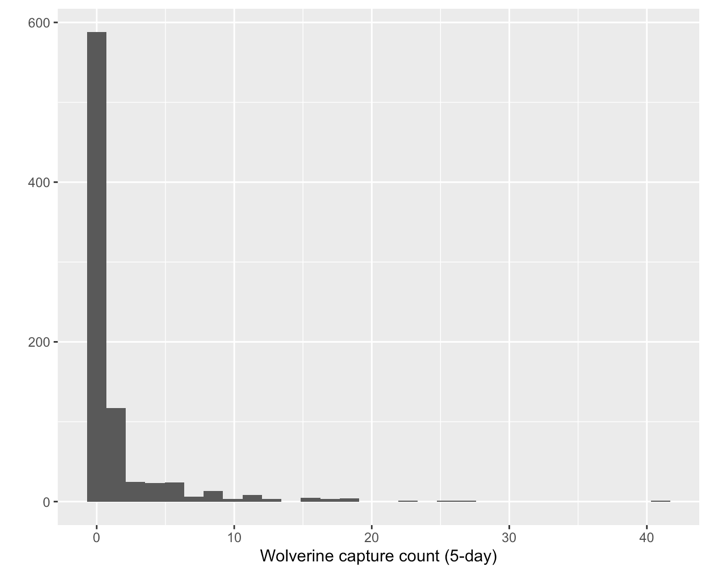

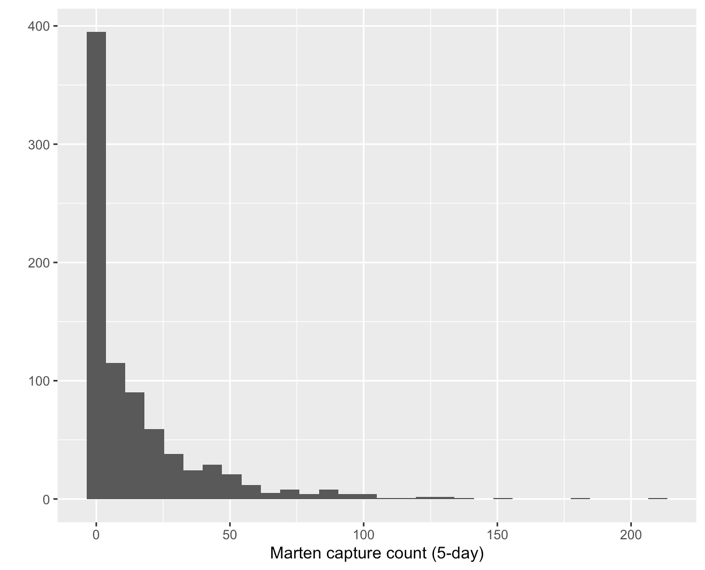

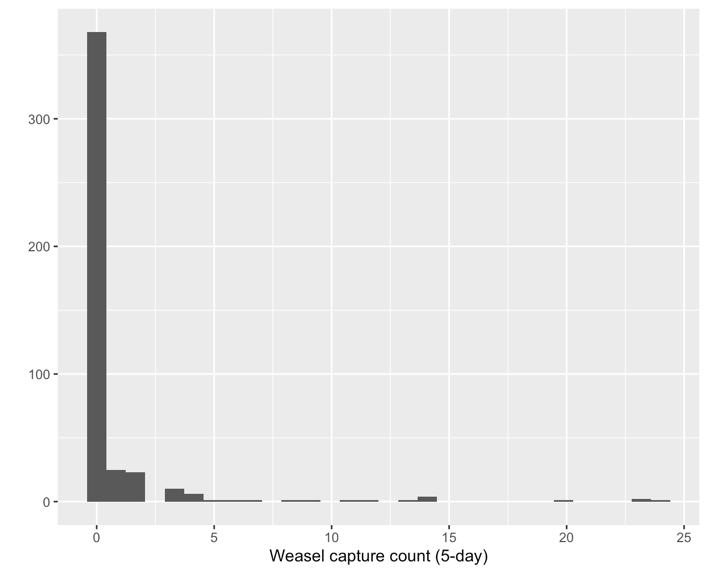


**Figure S1**: Number of independent captures for focal species over 5-day sampling intervals: wolverine (2006-2008, n = 1080), marten (2006-2008, n = 12599) and weasel (2007-2008, n = 342) in the WWP. Percent total sampling occasions that resulted in a zero are: wolverine (71.19 %), marten (34.14 %) and weasel (82.14 %).

**Table S1:** Kendall rank correlation for species pairings. P-values indicate that the Tau statistic is significantly different than 0, where -1 indicates a strong negative correlation and 1 indicates a strong positive correlation.

| **Kendall rank correlation test** | **Tau statistic** | **p-value** |
| --- | --- | --- |
| Wolverine-Marten | 0.083 | 0.003* |
| Wolverine-Weasel | 0.023 | 0.467 |
| Wolverine-Lynx | 0.061 | 0.062 |
| Marten-Weasel | -0.148 | 4.221e-07* |
| Marten-Lynx | -0.119 | 6.029e-05* |
| Weasel-Lynx | 0.02 | 0.558 |

**Table S2:** Habitat character classifications for examination of mesocarnivore space use in the Willmore Wilderness Area, derived from the ASLC dataset (ASLC, 2016). Resulting raster resolution equal to 25m. Among all camera sites, only two (sites 8 and 66) fell under shrub classifications, specifically, open upland shrub and shrubby wetland. Since both these are relatively open shrub dominated habitats, we included these in the open classification.

| **Habitat Character** | **Alberta Satellite landcover classifications** |
| --- | --- |
| **Open** | Open fir, open black spruce, open pine, open Engelmann/white spruce, open undifferentiated coniferous, tree dominated clear cut, fescue grassland, closed upland shrub, open upland shrub, Emergent wetland, Graminoid wetland, Shrubby wetland, Undifferentiated wetland, Black spruce bog, water (lakes), Permanent ice and snow, no Data, cloud/haze blocking, undifferentiated burn |
| **Closed Forest** | closed black spruce, closed pine, closed fir, closed Engelmann/White spruce, closed undifferentiated coniferous, Fir dominated coniferous, Black Spruce Dominated coniferous, Pine dominated coniferous, White spruce dominated coniferous, closed aspen/balsam popular/birch, closed deciduous dominated mixedwood, closed mixedwood |

**Table S3:** Model selection for mustelid count data regression models based on Pearson’s dispersion statistic for overdispersion and Vuong test statistic to assess zero-inflation. ZINB were found to be the best suited models for marten and wolverine datasets, whereas a NB-GLM was more suitable for the weasel distribution. In the case of weasel count data, the negative binomial general linear model resulted in a comparable dispersion statistic to that of the ZINB model. The Vuong statistic revealed that the NB-GLM performed better for weasel count data and thus represented a more suitable model for these data. Percent total sampling occasions that resulted in a zero are: wolverine (71.19 %), marten (34.14 %) and weasel (82.14 %).

| **Focal Species** | **Model Structure** | **Pearson’s Dispersion Statistic** | **Vuong Non-Nested Test**  **Statistic (VS)** |
| --- | --- | --- | --- |
| Weasel | GLM-P | 7.016 | AIC-corrected VS: 1.68  GLM-NB > ZINB  p-value 0.05 |
|  | GLM-NB | 0.919 |  |
|  | ZIP | 1.55 |  |
|  | ZINB | 0.967 |  |
| Marten | GLM-P | 31.73 | AIC-corrected VS: - 5.88  ZINB > GLM-NB  p-value 2.06 e-09 |
|  | GLM-NB | 0.837 |  |
|  | ZIP | 5.812 |  |
|  | ZINB | 0.999 |  |
| Wolverine | GLM-P | 8.91 | AIC-corrected VS: - 2.13  ZINB > GLM-NB  p-value 0.02 |
|  | GLM-NB | 0.926 |  |
|  | ZIP | 1.901 |  |
|  | ZINB | 0.958 |  |

**Table S4:** Summary statistics for TTE_r_ and TTE_obs_ distributions for all species pairings (minimum, , median, mean and maximum values). Results of the Wilcoxon rank sum test are given where, alternative hypothesis: true time-to-event is not equal to what would be expected at random. The p-value of the test is less than the significance level alpha = 0.05.

| **Species Pair: SpA (SpB)** | | | | | | | | |
| --- | --- | --- | --- | --- | --- | --- | --- | --- |
| **Random TTE distribution (TTEr)** | | | | **Observed TTE distribution (TTE_obs_)** | | | | **Mann–Whitney U test** |
| **(A) Wolverine (Marten): TTE_r_ n = 475.2, TTE_obs_ n = 372** | | | | | | | | |
| Min | Median | Mean | Max | Min | Median | Mean | Max | Mann–Whitney U test |
| 0.00 | 6.79 | 15.90 | 117.15 | 0.00 | 3.49 | 9.80 | 97.45 | W = 136618,  p = 5.90 e-10 |
| **(B) Wolverine (Weasel): TTE_r_ n = 44.8, TTE_obs_ n = 30** | | | | | | | | |
| Min | Median | Mean | Max | Min | Median | Mean | Max | Mann–Whitney U test |
| 0.55 | 19.15 | 22.18 | 83.70 | 0.20 | 6.52 | 12.34 | 73.03 | W = 962,  p = 1.95 e-3 |
| **(C) Marten (Wolverine): TTEr n = 144, TTEobs n = 357** | | | | | | | | |
| Min | Median | Mean | Max | Min | Median | Mean | Max | Mann–Whitney U test |
| 0.550 | 21.500 | 29.427 | 95.483 | 0.150 | 3.433 | 10.182 | 103.850 | W = 40038,  p = 1.46 e-22 |
| **D) Marten (Weasel): TTE_r_, n = 27.2, TTE_obs_ n = 39** | | | | | | | | |
| Min | Median | Mean | Max | Min | Median | Mean | Max | Mann–Whitney U test |
| 1.10 | 21.50 | 29.43 | 103.43 | 0.00 | 5.02 | 12.05 | 75.90 | W = 780,  p = 7.55 e-4 |
| **(E) Weasel (Marten): TTE_r_ n = 173.6, TTE_obs_ n = 39** | | | | | | | | |
| Min | Median | Mean | Max | Min | Median | Mean | Max | Mann–Whitney U test |
| 0.07 | 4.48 | 6.90 | 44.68 | 0.53 | 4.68 | 7.46 | 60.70 | W = 3282,  p = 0.75 |
| **(F) Weasel (Wolverine): TTE_r_ n = 66.4, TTE_obs_ n = 27** | | | | | | | | |
| Min | Median | Mean | Max | Min | Median | Mean | Max | Mann–Whitney U test |
| 0.17 | 6.41 | 11.31 | 61.27 | 0.80 | 6.47 | 11.70 | 60.93 | W = 884.5,  p = 0.96 |
